# Anxiety-Like Behavioural Inhibition Is Normative under Environmental Threat-Reward Correlations

**DOI:** 10.1371/journal.pcbi.1004646

**Published:** 2015-12-09

**Authors:** Dominik R. Bach

**Affiliations:** 1 Department of Psychiatry, Psychotherapy, and Psychosomatics, University of Zurich, Zurich, Switzerland; 2 Neuroscience Center Zurich, University of Zurich, Zurich, Switzerland; 3 Wellcome Trust Centre for Neuroimaging, University College London, London, United Kingdom; Imperial College London, UNITED KINGDOM

## Abstract

Behavioural inhibition is a key anxiety-like behaviour in rodents and humans, distinct from avoidance of danger, and reduced by anxiolytic drugs. In some situations, it is not clear how behavioural inhibition minimises harm or maximises benefit for the agent, and can even appear counterproductive. Extant explanations of this phenomenon make use of descriptive models but do not provide a formal assessment of its adaptive value. This hampers a better understanding of the neural computations underlying anxiety behaviour. Here, we analyse a standard rodent anxiety model, the operant conflict test. We harvest Bayesian Decision Theory to show that behavioural inhibition normatively arises as cost-minimising strategy in temporally correlated environments. Importantly, only if behavioural inhibition is aimed at minimising cost, it depends on probability and magnitude of threat. Harnessing a virtual computer game, we test model predictions in four experiments with human participants. Humans exhibit behavioural inhibition with a strong linear dependence on threat probability and magnitude. Strikingly, inhibition occurs before motor execution and depends on the virtual environment, thus likely resulting from a neural optimisation process rather than a pre-programmed mechanism. Individual trait anxiety scores predict behavioural inhibition, underlining the validity of this anxiety model. These findings put anxiety behaviour into the context of cost-minimisation and optimal inference, and may ultimately pave the way towards a mechanistic understanding of the neural computations gone awry in human anxiety disorder.

## Author Summary

Behavioural inhibition is observed in situations of anxiety, both in animals and humans. In some situations, it is not clear how it minimises harm or maximises benefit for the agent, and can even appear counterproductive. This prevents an understanding of the underlying neural computations. Here, we furnish the first formal assessment of its adaptive value in a controlled anxiety model, and confirm predictions in four experiments with humans. Results may suggest a neural implementation that relies on online cost minimisation. This finding could afford a better understanding of human anxiety disorder and the underlying neural computations.

## Introduction

Rodent models of human anxiety commonly involve a conflict between approach and avoidance [1–4], as exemplified in the Elevated Plus Maze [5–7], Open Field test [8], operant conflict tests [9, 10], or novelty-suppressed feeding test [11]. Behavioural inhibition is a core anxiety-like readout in these models, defined for example as a delay to initiate approach [11], and reduced by anxiolytic drugs [1]. Behavioural inhibition is also observed in non-human [12] and human primates [13]. Hippocampus lesions reduce anxiety-like behaviour in rodents [1, 14, 15] and humans [13] alike which suggests a neural implementation that is conserved across species. Extant theories assume that behavioural inhibition arises because of time requirements in the decision-making process: in one model because the animal uses that time to collect information about the situation (risk assessment) [1, 16], and in another suggestion because the decision whether to approach or to avoid is difficult [7]. Yet, a formal analysis of its adaptive value is lacking, and this impedes understanding the neural implementation of this behaviour. Here, we provide a normative explanation for behavioural inhibition in the framework of Bayesian Decision Theory (BDT) [17] by showing that it is the cost-minimising strategy in temporally correlated environments. Experimentally, we then demonstrate behavioural inhibition in humans, with a pattern that cannot be explained by previous accounts but is consistent with model predictions and may suggest a neural implementation based on goal-directed cost minimisation. Crucially, behavioural inhibition as measured in our task is related to individual anxiety scores, and this independently confirms the validity of the experimental anxiety model.

**Fig 1:**
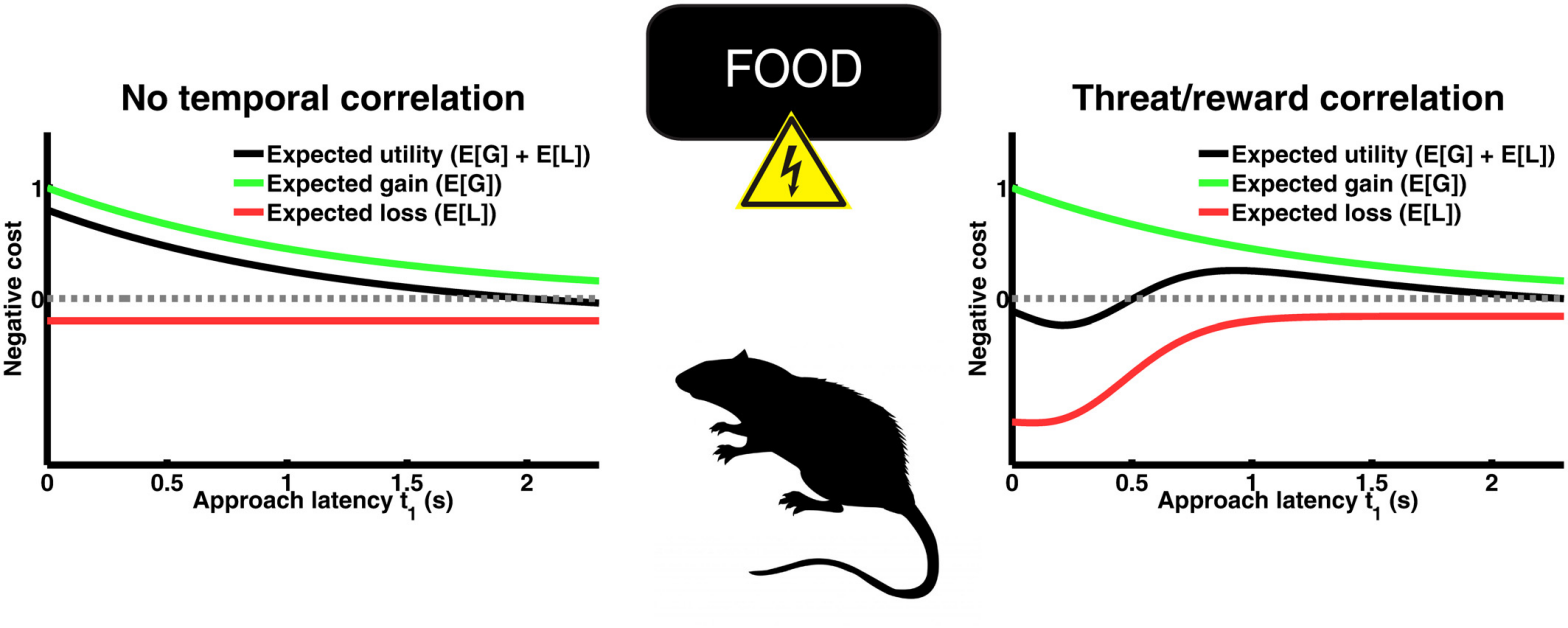
Two scenarios for a rodent conflict test. An animal is rewarded with food pellets for approaching a pellet dispenser, but there is a possibility of being punished by an electric shock. In scenario 1, the probability of threat is constant over time (red line) while the probability increases over time that the food pellet is withdrawn (green line). Expected utility, or negative expected loss, is maximal if the animal approaches the dispenser as quickly as biologically possible. In scenario 2, the threat probability is initially very high and decreases afterwards. This reflects naturally occurring temporal relations between predatory threat and reward. In this scenario, it is cost-minimising to move somewhat later (see Model and Methods for proofs, and S1 Text for the choice of parameters in these simulations).

Consider a rat in an operant conflict test in which it is trained to obtain a food pellet after a discriminative cue (Fig 1). On a proportion of trials, it will receive an electric shock together with the food pellet. Access to the food pellet is withdrawn if the animal does not respond within some time interval. In this scenario, the animal must make two decisions: whether or not to approach and collect the reward (the action), and if yes, when to approach (the approach latency, also termed response vigour [18, 19]). It should take the approach action if the utility of approach is expected to be positive. In this case, the animal should choose its approach latency in order to maximise expected utility. To do so, BDT mandates that it computes the probabilities of gaining the food pellet and getting the electric shock as functions of approach latency, and combines them with loss functions, to maximise its gain. Crucially, the probability functions rely exclusively on the animal’s (subjective) prior probabilities because there is no current indicator of the action outcome (i. e. no likelihood).

**Fig 2:**
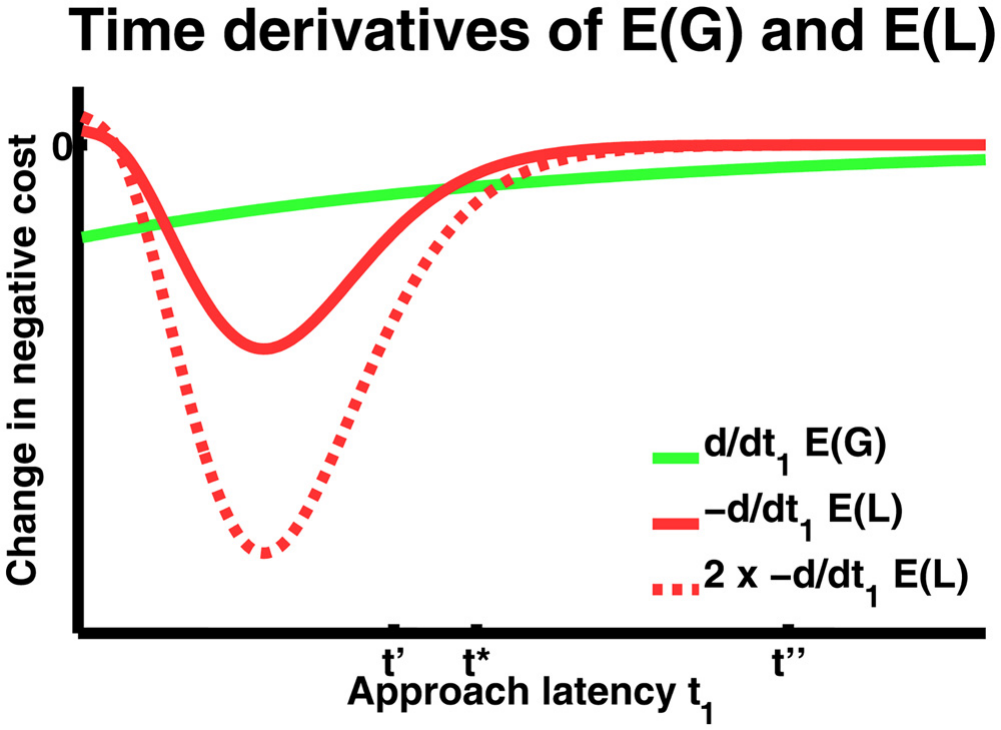
Finding the approach latency that maximises expected utility. First time derivatives of expected gain *E*(*G*) and negative expected loss −*E*(*L*). Under assumptions 7–8 (Model and Methods), the two curves must cross at least once, and that means there must be at least one stationary point. At least one of these stationary points is a maximiser. Crucially, the dotted line shows the impact of a small increase in *L* or a scaling of *p*(*L*). As one can see here, this will shift the optimal approach latency to the right, i. e. to later time points. The argument is formalised using Taylor series (see Model and Methods).

If the animal knew the objective task statistics (i. e. that probability of reward decreases over time, probability of shock is constant over time), its optimal decision would be to approach immediately after a reward appears (scenario 1, Fig 1 left). However, the animal has to learn these statistics. Before making a first response, it will rely on priors formed in other environments. Food availability is spatio-temporally coupled with predatory threat for many species [20, 21]. In biological terms, small-scale temporal correlations reflect a situation in which a predator is alerted by the occurrence of a reward that his prey species is interested in, and loses interest after waiting in vain for some time. Prey can exploit this environmental dependency to predict predatory threat. If the animal’s initial prior encodes a small-scale temporal correlation of threat and food reward, we will show that behavioural inhibition arises as cost-minimising strategy. This is illustrated in Fig 1 (scenario 2, right) and Fig 2.

Scenario 1 mandates immediate approach, which is biologically impossible if the food appears at unknown time points. Hence, an animal will show non-zero response latency in both scenarios 1 and 2, i. e. regardless of its priors. In order to distinguish between these two scenarios, we can analyse the impact of threat magnitude on optimal approach latency. It turns out that the cost-minimising approach latency in scenario 2 but not in scenario 1 depends on threat magnitude. This mathematical insight affords an empirical distinction between scenarios 1 and 2.

## Results

### Model results

The model is formulated in general terms and specifies the optimal approach latency $t_{1}^{*}$ as a local maximiser, i.e. by finding roots of the cost function derivative (eq 5). The first crucial finding is that non-zero approach latency, i.e. behavioural inhibition, is normative under very general priors about the temporal evolution of threat. Behavioural inhibition does not depend on the precise functional form of the prior and arises for all priors with a half-life of reward that is longer than the half-life of threat. This fact is illustrated in Fig 2 by using the same example prior as in Fig 1.

The value of the optimal approach latency in scenario 2, $t_{1}^{*}$, can only be predicted when the temporal evolution of *P*_*L*_ is known, which is not the case in an operant conflict task. Therefore, if we empirically measure a non-zero approach latency *t*_1_, we would not know whether this is due to biological constraints that delay the optimal response in scenario 1, or due to an non-zero optimal approach latency in scenario 2. We therefore analysed the impact of small changes in the parameters on the optimal approach latency in scenario 2. It turns out that small changes in potential loss, or overall threat probability, must increase the optimal approach latency, i.e. delay the response further (eq 8). This distinguishes scenario 2 from a model with time-varying biological constraints. For example, a time-dependent motor cost can also lead to a non-zero maximiser $t_{1}^{*}$, but here the optimal approach latency depends only on the gain, not on changes in threat probability or loss magnitude (S1 Text).

### Experimental confirmation

**Fig 3:**
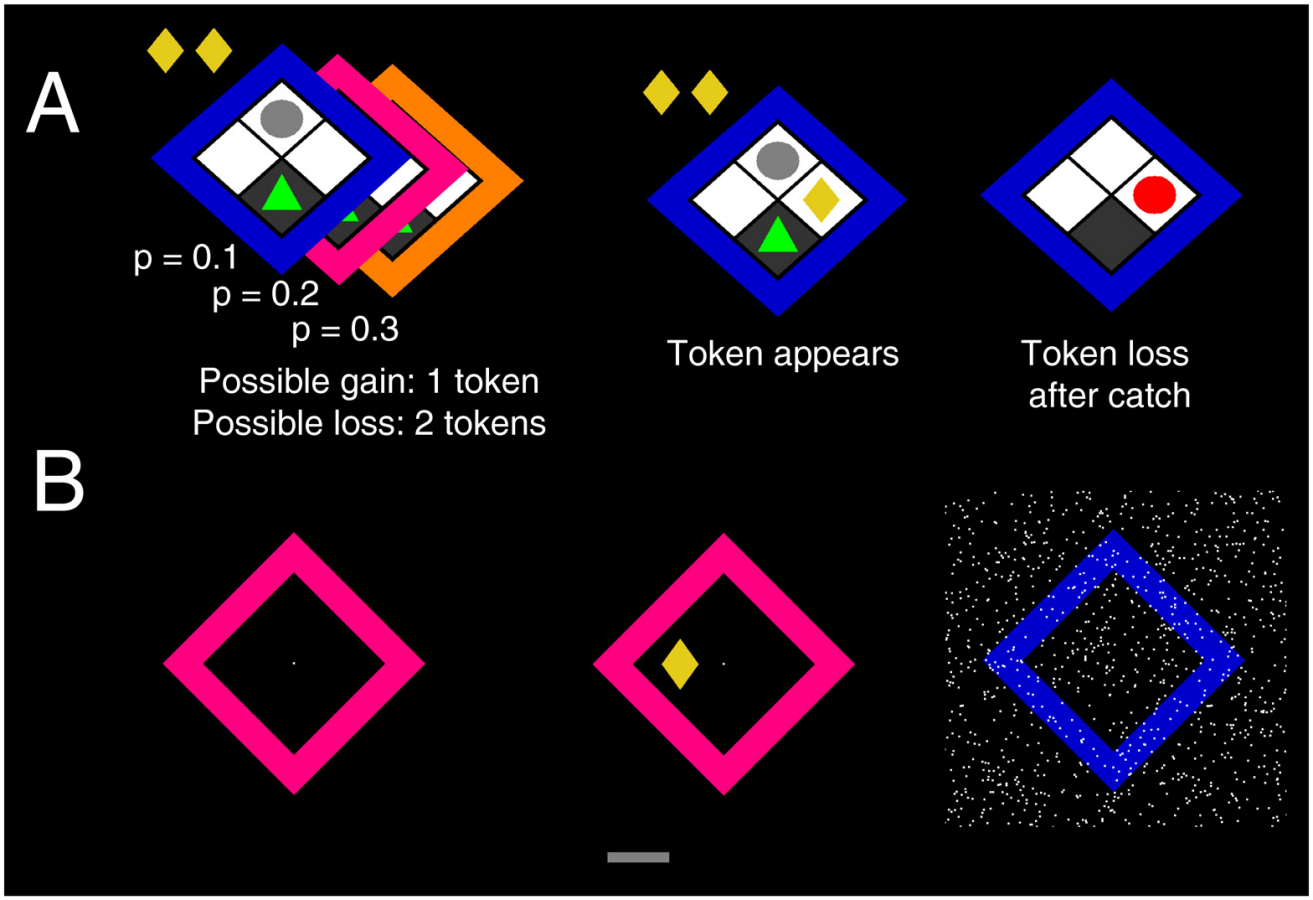
Human approach-avoidance conflict model. A: In experiments 1, 2 and 4, a human player (green triangle) rests in a safe place on a 2×2 grid, opposite a “sleeping predator”(grey circle). On each epoch, 6 successive reward tokens appear on the remaining two grid blocks at random time points. Once they have appeared on the grid, the time until they disappear is exponentially distributed. The player can press a key (experiments 1–2) or move a joystick (experiment 4) to collect these tokens which accumulate over any given epoch. At any time during the game, the predator becomes active with constant probability, but once active it will only reveal itself if the player is currently outside the safe place. If the player is caught by the predator, it loses all tokens already collected in this epoch, and no more new tokens appear. Magnitude of potential loss therefore corresponds to the number of already collected tokens. Threat level, defined as the wake-up rate, is different for the three predators. This wake-up rate is signalled by different colours, and tailored to result in a wake-up probability of p = 0.1, p = 0.2, or p = 0.3 if the player stays outside the safe place for 100 ms. Participants played 270 epochs (experiment 2: 210 epochs), thus making up to 1620 choices. B: In experiment 3, the task statistics were the same as in experiment 1 but the graphical set up and cover story were entirely different. The player is required to move a virtual “lever”(grey bar at the bottom) to obtain tokens, which can be removed if “static interference” occurred during lever movement.

We experimentally tested predictions from this mathematical model in experiment 1 with n = 20 human participants. Similar to a previous approach in humans [13], we modeled an operant conflict test as virtual computer game, with objective statistics according to scenario 1 (Fig 3). The player is tasked to collect tokens under threat of being caught by a “predator”which can catch the player to remove previously collected tokens. In this task, threat probability corresponds to the wake-up rate of the predator, termed threat level, and magnitude of potential loss corresponds to the number of already collected tokens, termed potential loss. The wake-up probability of the predator is constant over time, and the player has no possibility to escape once the predator is active. Tokens disappear according to an exponential distribution. Under the objective task statistics, optimal approach latency is independent from threat probability or magnitude, and should be minimised as much as biologically possible. However, if the human player uses (subjective) priors as in scenario 2, approach latency should increase as threat probability or magnitude of potential loss increases. Importantly, tokens appear sequentially, such that a decision whether and when to collect the next token can be made before it appears. Hence, decision difficulty should not delay responses once the token appears.

**Fig 4:**
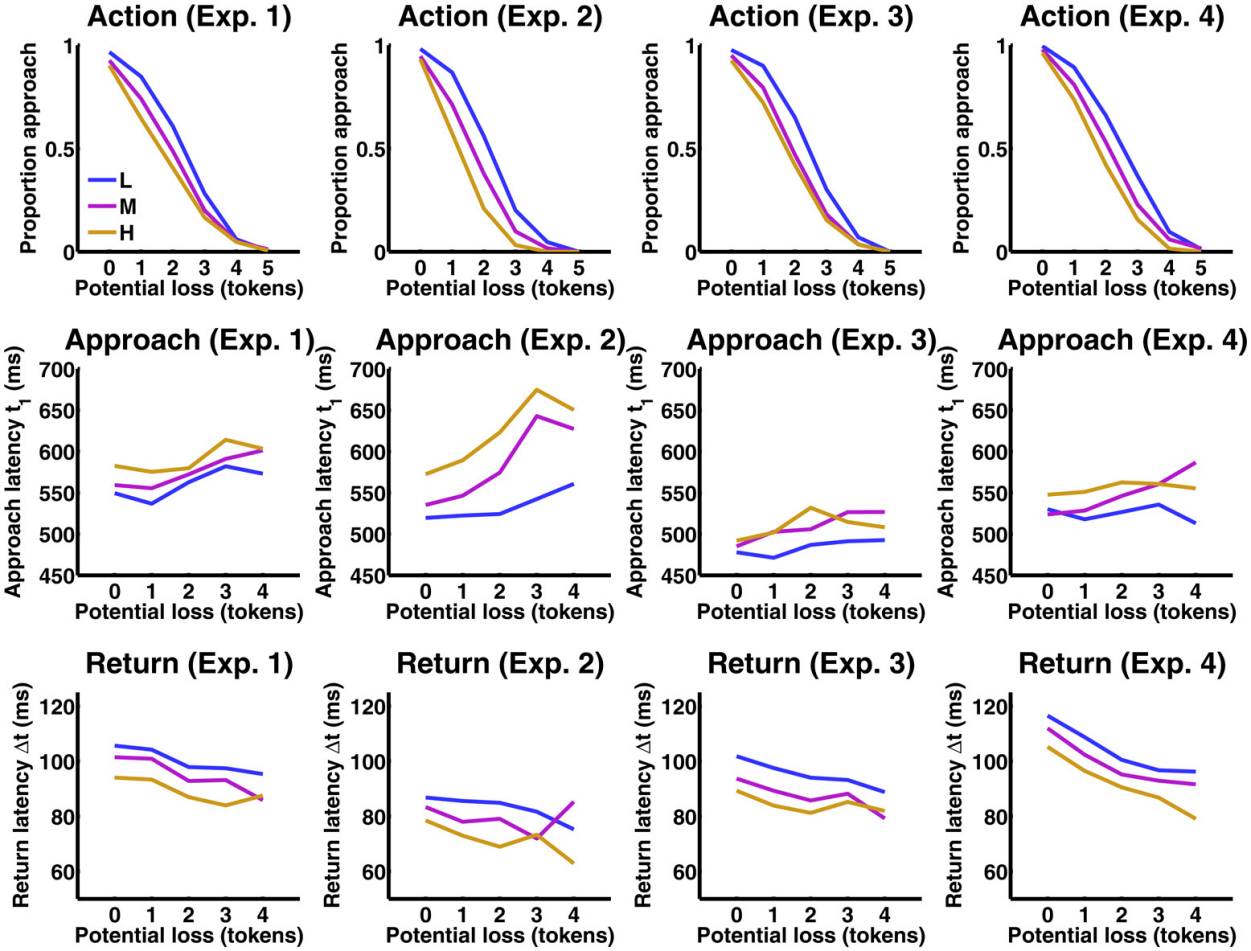
Results from experiments 1–4. The figure shows responses to the possibility to collect the nth token after already having collected (n—1) tokens which constitutes the potential loss. L: low threat. M: medium threat. H: high threat. Action: Percentage of epochs in which the player chose to collect at least the nth token. One can see that on the first token, i. e. when there is no potential loss involved, players almost always approach. After collecting increasingly many tokens, approach choices are reduced, and they are also reduced by higher threat level, (i. e. probability of loss). Approach and return latencies: Because the players rarely approached after collecting 5 tokens, approach latency is only shown up to a potential loss of 4 tokens. As the data are unbalanced, mean approach latencies were estimated in a linear mixed effects model (see Model and Methods). Approach latencies are increased both by increasing potential loss (i. e. number of already collected tokens) and by increasing threat level (i. e. probability of loss). The reverse pattern is seen for return latencies.

Fig 4 shows that participants were less likely to approach a token if threat level or potential loss were higher, as would be expected by rational agents. Strikingly, when they made a choice to approach the token, approach latency increased linearly both with increasing threat level (Linear Mixed Effects Model, F(1, 15485) = 21.9, p < 1e-5) and with increasing potential loss (F(1, 15485) = 19.8, p < 1e-5). This is the cost-minimising strategy when using priors according to scenario 2. It cannot be explained under scenario 1. Because there is no information to be gained from approach delay, this behaviour cannot reflect risk assessment [16]. Also, because a decision whether or not to go can be made before the token appears, behavioural inhibition is not explained by invoking decision difficulty [7].

We were concerned that players might delay their approach to improve performance of going into the correct left/right direction, or to minimise the time they spent outside the safe place. Players made on average 97.2% correct left/right responses. Correctness did not depend on threat level or potential loss (both p >.25), and also not on the variation in approach latency (i. e. after subtracting the average approach latency for each combination of threat level/potential loss/player, p >.10). Hence, increasing approach latency did not improve performance. Further, analysis of the variation in response and return latency (over and above impacts of threat level and potential loss, i. e. after subtracting the average approach latency for each combination of threat level/potential loss/player) revealed that an increase in approach latency of 100 ms lead to a decrease in return latency of 0.9 ms (t(12645) = -3.9, p <.0005). Although significant, the impact of this relation on action outcomes (i. e. probability of getting caught) is negligible. Further, time-dependent motor costs cannot explain the pattern of behavioural inhibition: a model including these costs predicts a dependency of the approach latency on potential loss but not on threat level (S1 Text). Approach latency distributions offered no evidence for a drift-to-threshold decision process [22] as reason for behavioural inhibition (S1 Text). Finally, results were replicated in a similar experiment 2, which balanced the colour-threat associations across participants. Again, approach latency increased linearly both with increasing threat level (F(1, 11808) = 38.9, p < 1e-10) and with increasing potential loss (F(1, 11808) = 7.9, p <.01).

To summarise, human behaviour in this task is predicted under subjective priors encoding a temporal coupling of threat and reward. How is this behaviour instantiated neurally? One possibility is that humans implement BDT in goal-directed online computations, in order to optimise their outcome on each trial. On the other hand, there may be a hard-wired (“Pavlovian”) inhibition mechanism invoked by predator/prey scenarios that reflexively delays actions whenever detecting approach-avoidance conflict, without any online considerations of future outcomes [23]. This mechanism would nevertheless invoke behaviour that is optimal in many natural environments in which reward/threat associations occur [20, 21]. Pavlovian biases are often thought to depend on primary reinforcers [23]. In our task, all reinforcement is financial, but the graphical setup strongly frames this as “predation” which could invoke an association with a primary reinforcer. Hence, in experiment 3 with n = 22 human participants, we controlled whether behavioural inhibition depends on a predation scenario.

The task had precisely the same statistics and required the same key presses as in experiment 1 but with an entirely different graphical setup and explanation such as to avoid any association with biological predation (Fig 3B). Human players were instructed to collect tokens by moving a virtual left/right “lever” back and forth by pressing keys, but that their action might be corrupted by “static noise”, and then they would lose all previously collected tokens. As in experiment 1, approach latency increased linearly with increasing threat level F(1, 17398) = 21.9, p < 1e-9) and with increasing potential loss F(4, 17398) = 13.0, p < 1e-3). Comparing data from both experiments in one statistical model revealed no difference in the impact of threat level or potential loss (interaction with experiment factor, both p >.20, see S1 Text). Strikingly, however, approach latency was considerably longer in experiment 1 (Experiment 1: 576 ms; experiment 3: 501 ms; main effect of experiment: F(1, 32902) = 587.9; p < 1e-128). This demonstrates that behavioural inhibition does not depend on the prospect of predation, but that the amount of inhibition depends crucially on the particular environment, consistent with a goal-directed, online implementation of BDT.

A possibly hard-wired, Pavlovian, inhibition mechanism might occur at a late motor stage. In a final experiment 4, we tested whether a possible inhibition mechanism suppresses or delays motor responses. To this end, experiment 1 was repeated, and human players made their responses with a joystick. They would collect the token if the joystick was moved beyond a certain threshold. As in previous experiments, overt approach latency increased with threat level and potential loss (Fig 4, S1 Text), and the same was found for the latency of motor initiation (S1 Text). If behavioural inhibition suppressed already initiated movements, one would expect more sub-threshold movements as threat level and potential loss increased, and as overt movements decreased, which was not the case. Instead, both overt and sub-threshold movements were inhibited as threat level/potential loss increased (S1 Text). Motor execution after response initiation was not impacted by threat level, and was faster (not slower) for higher potential loss (S1 Text). This demonstrates that behavioural inhibition in this task is not due to interference during motor execution and reflects action planning, again consistent with a goal-directed implementation of BDT.

Rodent conflict tests are often regarded as anxiety tests by face validity of the observed behaviour, or because of the specific behavioural alterations elicited by anxiolytic drugs [5]. However, it is not entirely clear that this behaviour relates to subjective feelings of anxiety in humans, experimentally often elicited by procedures involving social evaluation [24] which are however unaffected by anxiolytic drugs [25]. Hence, we tested whether subjective feelings of anxiety, recorded before the experiment using a standard questionnaire [26], predicted behavioural inhibition during our task. Because of the small baseline variation in anxiety, we increased power by combining data from experiments 1 and 2 that used the same graphical setup and response modality. Momentary (“state”) anxiety had no influence on approach latencies. Trait anxiety however, as a personality measure, impacted both the effect of threat level (interaction: F(2, 22928) = 10.1, p < 5e-4) and potential loss (F(4, 22928) = 3.9, p <.005) on approach latency while leaving overall approach latency unaffected. This finding is consistent with an idea that individuals with higher trait anxiety scores use a different prior threat probability function than individuals with lower trait anxiety. Note that in our model there is no linear relation between the prior and the approach latency; instead the impact of changing threat level/potential loss on approach latency depends on the precise curvature of the prior threat probability at the optimal approach latency. In line with this, non-linear interaction terms of trait anxiety with threat level and approach latency contributed to the effect of trait anxiety.

### Comparison with the model

**Fig 5:**
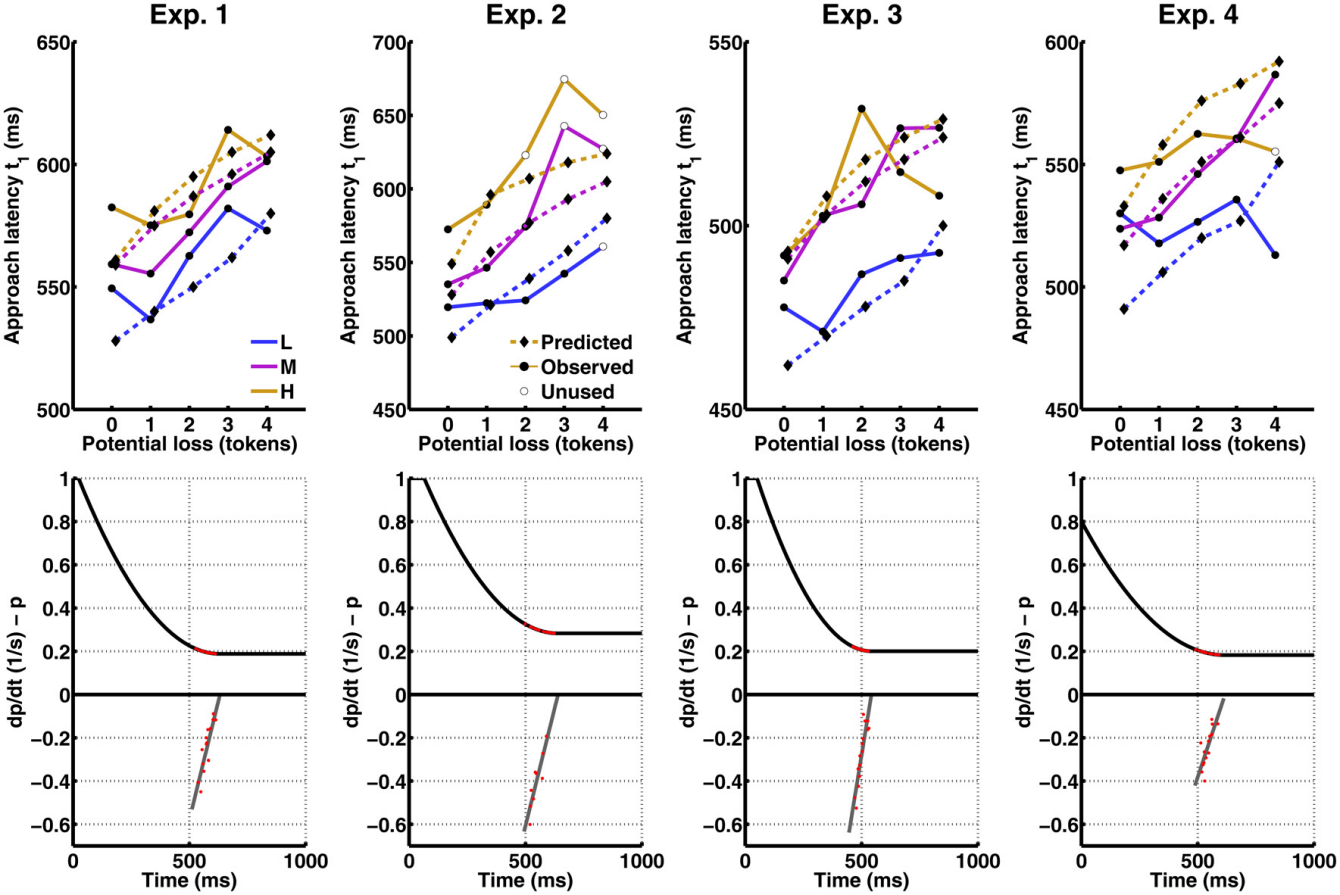
Comparison of model predictions with observed approach latencies. Upper panels: Predicted and observed approach latencies. Empty dots depict data points unused for the estimations (see Model and Methods). Lower panels: Reconstructed prior derivative (grey) and prior distribution (black). The prior derivative is scaled by the current catch rate and multiplied with current potential loss to derive the derivative of the expected loss (red curves in Fig 2). Red dots on the prior derivative depict data points used for the linear fit. Red dots on the prior depict range of predicted approach latencies.

Approach latency patterns across 4 experiments were qualitatively consistent with model predictions. In particular, a monotonic dependency of approach latency on threat level and potential loss is in keeping with eq (8). Additionally, we sought to quantitatively compare approach latencies to the model. This addresses whether a prior distribution *P*_*L*_ exists that can explain the observed data. The model’s quantitative predictions crucially depend not only on *P*_*L*_ but also on the internal representation of the gain probability, *P*_*G*_, and the internal utility of each loss and gain. To avoid overfitting the model to our data, we constrained our comparison by assuming that *P*_*G*_ is perfectly learned, that all values are weighted linearly, and that an ideal observer evaluates possible loss on each trial. For each experiment, this ideal observer model took as inputs the average number of collected tokens per threat level, the average (empirical) rate of getting caught per threat level, and the average rate of collecting a token when making an approach movement. After calculating the possible loss for each condition, we used eq (5) together with the observed approach latencies per condition, and the true values for *P*_*G*_, to compute the time derivative of the prior threat distribution. Fig 5 (lower panels) shows that these values were well approximated with a linear fit, a non-trivial observation that does not follow from the model or from the way of computing these values. The linear approximation for the derivative of the prior threat distribution was constrained to be negative (as per assumption 7), integrated, and a constant parameter added to achieve the average catch rate at the average approach latency across all conditions. The resulting prior threat distribution is shown in Fig 5 (lower panels) and is qualitatively similar across experiments, with the exception of exp. 3 where it reached a baseline more quickly. Finally, this 3-parameter prior was fed into the model (eq 5) to make quantitative predictions for approach latencies. Non-trivially, a local maximiser was found for all conditions in all experiments, and the predicted approach latencies approximated the monotonic trend observed in the data, while not accounting for experiment-specific kinks in the approach latency curves, as shown in Fig 5 (upper panels).

## Discussion

Behavioural inhibition is a core readout of rodent anxiety tests involving a conflict between approach and avoidance. This phenomenon has been explained with time requirements imposed by the decision process—time to gather further information (risk assessment [1, 16]), or time to complete a difficult decision [7]. In the current study, we provide an alternative explanation by analysing the adaptive value of this behaviour. We mathematically demonstrate that during operant approach/avoidance conflict, behavioural inhibition is the cost-minimising strategy in environments with small-scale temporal correlations of threat and reward [20, 21]. This mathematical model makes distinct predictions for an influence of threat level and potential loss on behavioural inhibition, which we experimentally confirm across 4 samples of human participants. The pattern of results cannot be explained by decision difficulty or risk assessment. Approach delay in our experiments depends on environment characteristics, but does not require the prospect of virtual predation, hence is not necessarily linked to primary reinforcers. Finally, this inhibition occurs before motor execution. It is therefore likely to arise from action planning and could possibly be instantiated by a goal-directed decision process as engendered in online cost minimisation according to BDT. Strikingly, human trait anxiety predicts behavioural inhibition in our study, confirming the validity of this operant conflict test as an anxiety model.

Quantitative comparison of our data with the model demonstrated that a threat prior can be constructed to explain the observed data. This prior was similar across experiments, and its simple shape is biologically plausible. While the predictions from that prior approximated a monotonic trend in the observed approach latencies, it did not predict experiment-specific deviations from this trend. Note however that our reconstruction of the prior assumed an ideal observer and linear utility functions for specifying the expected loss, and it was solely based on financial loss and did not include an any additional loss (a premium) for the fact that one gets caught. Estimating such valuation parameters is likely to improve the fit of the model and could be achieved in independent tasks. We note that the model fit does not prove that participants actually used this prior. Also, we did not aim to make participant-specific predictions. The range of approach latencies from which data points for reconstructing the prior can be sampled is much smaller than the time window searched for a maximiser. The present approach should therefore only be regarded as providing face plausibility to the model, not as a strategy to reconstruct the true priors that participants used. Future work will aim at estimating this prior from independent tasks and making specific quantitative predictions for optimal approach latency. Capitalising on different reward distributions, it is then possible to test the current model in which behavioural inhibition crucially depends on the interplay of reward and threat priors.

Across various tasks involving approach/avoidance conflict, anxiety-like behaviour intricately relies on the ventral hippocampus in rodents and humans, and is reduced by hippocampus lesions and anxiolytic drugs alike [1, 13–15]. Hippocampal oscillations in the theta range are a hallmark of rodent approach/avoidance conflict and also reduced by anxiolytics in frequency and amplitude [1, 2, 27]. Despite a wealth of electrophysiological and neuropharmacological knowledge, however, the function of the hippocampus in these tasks is hardly understood, and this is in stark contrast to theoretical models of hippocampal theta oscillations in memory and spatial navigation [28–31]. This lacuna might be partly due to the fact that behavioural objectives in many ethological approach/avoidance conflict tasks are complex and opaque, rendering an analysis of neural computations a difficult endeavour. In the current work, we took the approach of isolating individual actions and their outcomes from one another, and avoiding incentives relying exclusively on unknown internal processes such as the innate propensity to explore open spaces, harnessed in many rodent anxiety models. This strategy removed from our analysis all elements that could transform action planning into a multi-step problem [18, 19], and hence simplified it to an extent where an experimental confirmation was tractable. The series of experiments conducted here allows us to speculate about neural implementation where the empirical evidence is consistent with goal-directed planning although we cannot rule out a possible influence of pre-programmed (“Pavlovian”) biases that have evolved because they are adaptive in many natural environments. The current approach using isolated actions affords experimental analysis of prior belief distributions on threat-reward correlation and their neural implementation. It may thus enable a more detailed understanding of the neural circuits mediating anxiety-like behaviour. It can easily be complemented by an analysis of this behaviour in continuous time [18, 19], and thus, in more realistic foraging scenarios. Finally, the theoretical model is applicable to rodent behaviour and can be tested by finessing operant conflict tests in animals [32].

To summarise, we find that behavioural inhibition is a cost-minimising behaviour in many natural environments, and possibly instantiated neurally by online cost minimisation. Our finding puts anxiety-like behaviour into the context of optimal inference [17, 33, 34], action planning [18, 19, 35], and biological cost minimisation [36, 37]. Rather than being a somewhat irrational state of mind, anxiety is thereby rephrased as optimal behaviour under biologically plausible priors, which makes it accessible to the toolkit of computational analysis [38, 39]. It will be interesting to investigate whether individual differences in anxiety stem from variation in prior assumptions about environmental statistics—even implausible priors—, suboptimal use of such priors, or variation in updating such priors with experience. Ultimately, this research may pave the way towards a more mechanistic understanding of anxiety disorders.

## Models

### Model

All variables are explained in Table 1.

**Table 1:** Variables and symbols used in the model.

*Z*	Expected utility function being maximised
*G* > 0	Utility of a potential gain from collecting a reward (constant)
*L* < 0	Utility of a potential loss from receiving a punishment (constant)
*t*_0_ = 0	Time point at which the reward appears
*t*_1_ > 0	Time point at which the animal arrives at the reward (approach latency)
$t_{1}^{*}$	Optimal approach latency
*t*_2_ > *t*_1_	Time point at which the animal withdraws from the reward location
Δ*t* = *t*_2_ − *t*_1_	Time spent at the reward location (return latency)
*T* ∼ *Exp*(*λ*_1_)	Exponentially distributed random variable, indicating the time until the reward disappears, with mean 1/*λ*_1_
*x* ∼ *Poisson*(*λ*_2_)	Number of activation events of the punisher in the interval [*t*_1_, *t*_2_]
*P*_*G*_(*t*_1_) = *P*_*T* > *t*_1__	Prior probability of gain (twice differentiable wrt *t*_1_)
*P*_*L*_(*t*_1_, *Δt*) = *P*_*x* > 0_	Prior probability of loss (twice differentiable wrt *t*_1_). Note both pmfs take latencies as arguments, not as random variables.
*E*(⋅), *E*_*t*_1__(⋅)	Expectation over outcomes, independently evaluated at each approach latency *t*_1_

**Task structure**
1. Food pellets of equal nutritional value appear at random time points.
2. The duration during which the pellets can be collected is drawn from an exponential distribution with parameter *λ*_1_. Therefore:
  $$\begin{array}{c} P_{G}\left(t_{1}\right)=P_{T>t_{1}}=e^{-\lambda_{1}t_{1}}. \end{array}$$ (1)
3. The activation of the electric shock generator is a Poisson process. In other words: if the animal touches the pellet dispenser, it is equally likely to receive an electric shock at each point in time. Hence, according to the true task statistics (i.e. scenario 1):
  $$\begin{array}{c} P_{L}\left(\Delta t\right)=P_{x>0}=1-e^{-\lambda_{2}\Delta t}. \end{array}$$ (2)

**Assumptions**
**Biological assumptions**
1 Utility of electric shock and of food pellet are quantifiable, and do not change over the course of a trial.
2 All utilities combine linearly.
3 All utilities and the gain distribution are fully known to the agent.
4 There is no uncertainty on motor execution.
5 Preceding each pellet appearance, the animal makes a decision whether to go and collect the next pellet, and when to initiate its action.
6 The time spent at the dispenser is independent from the time of initial movement.

These assumptions bear on the structure of the model. The following assumptions change the behaviour of the model but do not constrain its structure.

**Assumptions for scenario 2**
7 The prior distribution embodies an assumption on threat probability: threat probability is high after a reward appears and decays afterwards:
  $$\begin{array}{c} \frac{\partial }{\partial t_{1}}P_{L}<0 \end{array}$$
  $$\begin{array}{c} \frac{\partial }{\partial t_{1}}E\left(L\right)>0 \end{array}$$
8 Also rewards decay:
  $$\begin{array}{c} \frac{\partial }{\partial t_{1}}E\left(G\right)<0 \end{array}$$
9 The decay rate of expected loss is at some point *t*′ higher than the decay rate of expected gain, and asymptotes quicker at a later time point *t*′′:
  $$\begin{array}{c} \left|\frac{\partial }{\partial t_{1}}E_{t^{\prime }}\left(L\right)\right|>\left|\frac{\partial }{\partial t_{1}}E_{t^{\prime }}\left(G\right)\right| \end{array}$$
  $$\begin{array}{c} \left|\frac{\partial }{\partial t_{1}}E_{t^{\prime \prime }}\left(L\right)\right|<\left|\frac{\partial }{\partial t_{1}}E_{t^{\prime \prime }}\left(G\right)\right|. \end{array}$$

#### Model structure

Bayesian decision theory (BDT) mandates minimising the expected subjective loss, i.e. maximising the expected utility. Because there is no sensory information on the outcome, the agent uses prior probabilities. Hence, the objective is to maximise

$$\begin{array}{c} Z\left(t_{1},\Delta t\right)=E\left(L\right)+E\left(G\right)=P_{L}\left(t_{1},\Delta t\right)L+P_{G}\left(t_{1}\right)G \end{array}$$ (3)

and the optimal approach latency is:

$$\begin{array}{c} t_{1}^{*}=\underset{t_{1}}{\arg\max}\;Z\left(t_{1},\Delta t\right). \end{array}$$ (4)

If a local maximiser $t_{1}^{*}$ exists, it constitutes a stationary point at which

$$\begin{array}{c} \frac{\partial }{\partial t_{1}}Z=\frac{\partial }{\partial t_{1}}E\left(G\right)+\frac{\partial }{\partial t_{1}}E\left(L\right)=0 \end{array}$$ (5)

because of assumption 6 (*t*_1_ and *Δt* are independent).

#### Optimising approach latency in scenario 1

If the prior distribution *P*_*L*_ reflects the true task statistics, the animal should move as quickly as biologically possible. Proof:

According to eq (2), the expected cost of moving to get the pellet is:

$$\begin{array}{c} E\left(L\right)=P_{L}L=L\left(1-e^{-\lambda_{2}\Delta t}\right). \end{array}$$

The expected gain from moving to get the pellet is decreasing over time, using eq (1):

$$\begin{array}{c} E\left(G\right)=P_{G}G=Ge^{-\lambda_{1}t_{1}}. \end{array}$$

Because of assumption 6 (*t*_1_ and *Δt* are independent):

$$\begin{array}{c} \frac{\partial }{\partial t_{1}}E\left(L\right)=0. \end{array}$$

$$\begin{array}{c} \frac{\partial }{\partial t_{1}}Z=\frac{\partial }{\partial t_{1}}E\left(G\right)=-G\lambda e^{-\lambda_{1}t_{1}}<0. \end{array}$$

Therefore, the maximiser for *Z* over any interval [*t*′, *t*′′] is $t_{1}^{*}=t^{\prime }$.

#### Optimising approach latency in scenario 2

Under assumption 7–9, a non-zero local maximiser $t_{1}^{*}$ for *Z* exists. Proof:

Consider *f* = ∂*Z*/∂*t*_1_. From assumptions 7–9, *f* (*t*′) > 0, *f* (*t*′′) < 0. Because *f* is continuous, *f* has one or several roots in [*t*′, *t*′′]. These roots are stationary points for *Z*. Because *f* is continuous, in at least one root $t_{1}^{*}$ it must change its sign from positive to negative, i. e.

$$\begin{array}{c} \frac{\partial^{2}}{\partial t_{1}^{2}}Z\left(t_{1}^{*}\right)<0. \end{array}$$

This shows that $t_{1}^{*}$ is a maximiser. The argument is also illustrated in Fig 2.

#### Impact of parameter changes in scenario 2

If $\frac{\partial }{\partial t_{1}}E\left(L\right)\neq 0$, and if this leads to a non-zero maximiser for *Z*, then increasing absolute potential loss, or the probability of loss increases the maximiser, and the agent should move later. Proof:

We approximate eq (5) with first order Taylor expansions around $t_{1}^{*}$, using eq (3):

$$\begin{array}{c} \frac{\partial }{\partial t_{1}}E_{t{}_{1}^{{}^{\prime }}}\left(G\right)≅G\frac{\partial }{\partial t_{1}}P_{G}\left(t{}_{1}^{*}\right)+\left[t_{1}^{{}^{\prime }}-t{}_{1}^{*}\right]G\frac{\partial^{2}}{\partial t_{1}^{2}}P_{G}\left(t{}_{1}^{*}\right) \end{array}$$ (6)

$$\begin{array}{c} \frac{\partial }{\partial t_{1}}E_{t{}_{1}^{{}^{\prime }}}\left(L\right)≅L\frac{\partial }{\partial t_{1}}P_{L}\left(t{}_{1}^{*}\right)+\left[t_{1}^{{}^{\prime }}-t{}_{1}^{*}\right]L\frac{\partial^{2}}{\partial t_{1}^{2}}P_{L}\left(t{}_{1}^{*}\right). \end{array}$$ (7)

Inserting eqs (6) and (7) into eq (5), we obtain:

$$\begin{array}{c} G\frac{\partial }{\partial t_{1}}P_{G}\left(t{}_{1}^{*}\right)+\left[t_{1}^{{}^{\prime }}-t{}_{1}^{*}\right]G\frac{\partial^{2}}{\partial t_{1}^{2}}P_{G}\left(t{}_{1}^{*}\right)+ \end{array}$$

$$\begin{array}{c} L\frac{\partial }{\partial t_{1}}P_{L}\left(t{}_{1}^{*}\right)+\left[t_{1}^{{}^{\prime }}-t{}_{1}^{*}\right]L\frac{\partial^{2}}{\partial t_{1}^{2}}P_{L}\left(t{}_{1}^{*}\right)=0. \end{array}$$

Hence:

$$\begin{array}{c} t_{1}^{{}^{\prime }}-t{}_{1}^{*}=-\frac{L\frac{\partial }{\partial t_{1}}P_{L}\left(t{}_{1}^{*}\right)+G\frac{\partial }{\partial t_{1}}P_{G}\left(t{}_{1}^{*}\right)}{L\frac{\partial^{2}}{\partial t_{1}^{2}}P_{L}\left(t{}_{1}^{*}\right)+G\frac{\partial^{2}}{\partial t_{1}^{2}}P_{G}\left(t{}_{1}^{*}\right)}. \end{array}$$ (8)

Obviously, the numerator in this equation is zero according to eq (5). The denominator is negative because $t_{1}^{*}$ is a maximiser:

$$\begin{array}{c} \frac{\partial^{2}}{\partial t_{1}^{2}}Z\left(t_{1}^{*}\right)=L\frac{\partial^{2}}{\partial t_{1}^{2}}P_{L}\left(t_{1}^{*}\right)+G\frac{\partial^{2}}{\partial t_{1}^{2}}P_{L}\left(t_{1}^{*}\right)<0. \end{array}$$

Now, if we slightly increase the absolute potential loss to *L*′ = *kL*, *k* > 1, the first term in the numerator of eq (8) becomes larger, and hence the numerator becomes positive, while for small changes, the denominator remains negative. Therefore:

$$\begin{array}{c} L^{\prime }<L\Rightarrow t_{1}^{{}^{\prime }}-t{}_{1}^{*}>0. \end{array}$$

Or in other words, for larger absolute potential loss, the optimal approach latency increases.

Analogously, an increase in the overall probability of loss may be assumed to be represented as

$$\begin{array}{c} P_{L}^{\prime }=kP_{L},\;k>1. \end{array}$$

Again, the first term in the numerator of eq (8) becomes larger. Therefore:

$$\begin{array}{c} P_{L}^{\prime }>P_{L}\Rightarrow t_{1}^{{}^{\prime }}-t{}_{1}^{*}>0. \end{array}$$

The argument is also illustrated in Fig 2.

### Experimental materials and methods

#### Ethics statement

The study protocol was in full accordance with the Declaration of Helsinki. All participants gave written informed consent after being fully informed about the purpose of the study. The study protocol, participant information, and form of consent, were approved by the competent research ethics committee (Kantonale Ethikkommission Zurich).

#### Participants

We recruited 20 participants for experiment 1 (8 female, mean age ± standard deviation: 24.5 ± 3.5 years), 21 participants for control experiment 2 which balanced colour-threat associations (11 female, mean age ± standard deviation: 23.9 ± 3.6 years), 22 participants for experiment 3 with a different graphical setup (11 female, mean age ± standard deviation: 25.9 ± 4.3 years), and 20 participants for experiment 4 with joystick responses (10 female, mean age ± standard deviation: 26.3 ± 4.8 years). State and trait anxiety were controlled with the German version of the state/trait anxiety inventory [26]. All participants expect one had values within 2 standard deviations around the reference sample mean (586 individuals between 15–29 years, both sexes). The combined sample had slightly lower state anxiety values than the reference sample (33.3 vs. 36.8, p <.001, Welch’s t-test) and slightly higher trait anxiety values (38.2 vs. 35.1, p <.001).

#### Experimental design

Each player played 270 (experiment 2: 310) successive epochs of the computer game. A sequence of 6 reward tokens appeared per epoch at random time points; the player could decide each time whether or not to collect. The human player was rewarded for the final outcome of one randomly drawn epoch at the end of the experiment. The interval between disappearance of one token and appearance of the next was the sum of the remaining presentation time from the last reward token (if collected before disappearance), a short constant interval of 500 ms, and a variable interval drawn from an exponential distribution with a mean of 1.25 s. Thus, neither action (Go or NoGo) was associated with an opportunity cost from the passage of time. The interval during which reward tokens were present and could be collected was also drawn from an exponential distribution with a mean of 1.25 s. A“sleeping predator” was waiting in the grid block opposite the human player. It became active in a homogenous Poisson process: if active and the human player inside the safe place, it deactivated itself. If active and the human player outside the safe place (regardless whether or not a reward token was present), it revealed itself and moved to the human player’s grid block. The human player was “eaten” and all previously collected reward tokens removed. Once the predator was active, the human player had no possibility to escape. In order to remove any time benefit (opportunity cost) from getting caught by the predator, the active predator staid visible on the screen for the remaining time of the epoch while the player was forced inactive. In control experiment 2, there were 210 epochs of the type as in experiments 1 and 3–4. In an additional interspersed 100 epochs, two robbers (high and low threat level) were signalled at the same time, and participants were told that they could not know which of the two robbers was present [40, 41]. Behaviour in these rounds was not different from behaviour in the medium threat condition and is not reported here.

**Parameters of reward and threat distributions**
1. Disappearance time of the token was drawn from an exponential distribution with mean $\lambda_{1}^{-1}=1.25\;s$. The variable part of the inter token interval was drawn from the same distribution.
2. For an assumed *Δt* = 100*ms*, the expected wake up probability for each move was set to *p* = 0.1, *p* = 0.2 or *p* = 0.3, for the three robbers respectively. Using eq (2), we see that *λ*_2_ = −ln(1 − *p*)/*Δt*. This results in event rates, for the three robbers, of *λ*_2,1_ = 1.0536/*s*, *λ*_2,2_ = 2.2314/*s* and *λ*_2,3_ = 3.5667/*s*.
3. The wake up events were independently determined in successive time bins of 20 ms duration.

#### Graphics and motor responses

For experiments 1, 2, and 4, a 2 × 2 grid in diagonal orientation was presented with ^~^4° visual angle on a standard LCD computer monitor. Quadratic grid blocks were white and separated by black gridlines. A coloured frame indicated the identity of the sleeping predator. The human player was represented by a green triangle, and the sleeping predator by a grey circle on the top grid block, which turned to red once it woke up and moved. The association of predator colours (blue, orange, purple) with threat levels was fixed in experiments 1, 3 and 4 and balanced across participants in experiment 2. Reward tokens, represented by yellow rhombi, could appear on the left and right grid blocks. The bottom grid block was dark grey, representing a “safe place” that the predator could not reach. Every collected token was placed in a row of collected tokens for this epoch above the grid, which was immediately removed once the player got caught, or when the epoch ended. The human player was controlled with the left/right cursor keys on a standard computer keyboard (experiment 1–3) and by diagonal forward movements of a joystick (experiment 4). The player could move to the left/right grid blocks and back at all times unless caught by the predator.

In experiment 3, a coloured frame, the same size as in the other experiments, surrounded a black square with a white point at the centre. There was no predator and no human player on the screen. Reward tokens could appear left/right of the centre point with the same appearance and in the same position as in the other experiments. In order to go and get a token, the human player had to turn a virtual “lever” by pressing the left/right cursor key, and turn it back into neutral position by pressing the opposite key. Hence, the same motor sequence was required as in the other experiments. If the lever was in non-neutral position, a slim grey bar under the play field indicated its position. Whenever the lever was in non-neutral position, “static noise” could appear on the screen, and this would remove the previously collected tokens. “Static noise” consisted of a 500 ms train of 3000 white dots, placed in random positions on the screen and changing their position at 30 Hz. For all experiments, head-screen distance was approximately 90 cm and at the participant’s discretion.

#### Data analysis—general

Data analysis followed a 3 (threat level) × 6 (potential loss) factorial design.“Threat level” corresponds to wake-up rate of the predator, and thus, to loss probability. “Potential loss” corresponds to number of already collected tokens. For each choice, we extracted the level of these two factors and the choice. In case of a Go choice, we also extracted the approach latency and return latency (i. e. time passed between moving out of the safe place, and moving back into it), and whether the response was in the correct direction (left/right). Approach latency was the primary dependent variable, while the other measures served as control measures. All data are necessarily unbalanced because the number of data points for each cell in the design depends on behavioural choices and on chance.

#### Data analysis—choice

Because the task design mandated successive collection of tokens, subsequent choices are dependent. As a result, choice data are both overspecified and incomplete on any given epoch. If an individual chooses, after obtaining *n* tokens in a given epoch, not to collect any more tokens, several data points might indicate a NoGo choice on the (*n* + 1)th token (here, *n* + 1 corresponds to the potential loss of *n* which repeats itself as the player does not collect tokens; it does not correspond to the sequence of presented tokens). But the individual also implicitly chose to not collect the (*n* + 2)th, (*n* + 3)th token, and so on, although no data points exist for these choices. In order to account for this, we reconstructed the player’s implicit choices. We created 6 data points for each epoch, corresponding to the possibility of collecting six tokens. For each of these six tokens, we recorded 1 if the individual chose to collect up to, or more than, this number of tokens on this epoch, and 0 if the individual chose to collect less than this number of tokens on this epoch. On epochs on which the player was caught, its choice of how many tokens to collect cannot be reconstructed—hence choices in these epochs were not analysed. The resulting choice data set was thus balanced with respect to potential loss. It was still unbalanced with respect to threat level because more epochs were missing (as the player got caught) at higher threat levels.

#### Data analysis—latencies

To avoid approach and return latency estimates being biased by extreme values, they were only analysed if they fell into response windows of 150 ms < approach latency < 2000 ms and 0 ms < return latency < 2000 ms, thus excluding, for the four experiments, 0.6%-1.5% of approach latencies and 0%-0.01% of return latencies. It is sometimes recommended to log-transform reaction times. All approach latency models were re-fitted with log-transformed latencies, replicating all significant findings. Most players rarely collected the 6th token such that some design cells were empty and the parameters could not be estimated reliably. Therefore, the 6th token was excluded for all approach/return latency and correctness analysis such that the resulting design was effectively a 3 (threat level) × 5 (potential loss) factorial design.

#### Data analysis—joystick responses

To analyse joystick responses in experiment 4, the joystick position was sampled in two dimensions at 50 Hz sampling rate. Overt responses were identified within the experimental software as joystick excursions above an absolute threshold in x- and y-direction, and the time point of overt responses was recorded online during the experiment. We computed offline the instantaneous vector modulus of the joystick position, multiplied by the sign of its projection onto the diagonal connecting player and token. Maximum excursion was extracted in the interval between token appearance and token disappearance for trials without overt responses, termed “NoGo trials” here. For “Go trials”, motor initiation latencies were identified as the first time after token appearance when three subsequent joystick samples moved into the quadrant the token appeared in. Response duration was the interval between motor initiation and overt response.

#### Data analysis—skin conductance responses

Skin conductance responses (SCR) in experiment 4 were analysed in a model-based approach, using a General Linear Convolution Model [42]. Each token appearance was modelled as a stick function, convolved with a canonical skin conductance response function and its time derivative [43]. Events were separated according to condition and correct Go/NoGo response, resulting in 36 regressors in the design matrix. Incorrect responses and trials in which the player got caught were collapsed in two additional regressors. Data were filtered with a unidirectional band pass filter (cutoff frequencies 0.05 Hz and 5 Hz) as recommended in [44] and down sampled to 10 Hz resolution. The model was inverted and estimates of sympathetic arousal computed as amplitude of the reconstructed response [44], for each participant and condition. SCR results are reported in S1 Text.

#### Statistical analysis

The lme4 package in the software R (www.r-project.org) was used for all statistical analysis as it provides meaningful parameter estimators for unbalanced data sets. Choice and correctness data were analysed using a generalized linear mixed effects model for binomial data as implemented in glmer. Approach and return latency were analysed using a linear mixed effects model as implemented in lmer. All models had the form

$$\begin{array}{c} \eta_{k}=\beta_{0}+\beta_{1}X_{1}+\beta_{2}X_{2}+\beta_{3}X_{3}+b_{k},\;k=1..n \end{array}$$

$$\begin{array}{c} b_{k}∼N\left(0,\sigma_{b}^{2}\right) \end{array}$$

where *β*_0_ is the group intercept, ***β*_1..3_** are the fixed effects parameter vectors for 3 threat levels, 6 or 5 token numbers, and their interaction, and *b*_*k*_ is the random subject intercept. ***X*_1..3_** encode the threat level, potential loss and interaction. This is equivalent to the R model formula

Y~threat*token+(1|subject).

For comparison of experiments 1 and 2, an additional fixed factor “experiment” and all interactions up to the 3rd level were added.

The linear predictor ***η*** is related to the data **y** through the identity link function for the approach and return latency data:

$$\begin{array}{c} y_{ijk}∼N\left(\eta_{ijk},\sigma^{2}I\right) \end{array}$$

and through the logit link function for the choice and correctness data:

$$\begin{array}{c} y_{ijk}∼B\left(1,\frac{1}{1+\exp\left(\eta_{ijk}\right)}\right). \end{array}$$

Fixed-effects F-statistics were extracted using the R function anova. P-values were calculated by using a (conservative) lower bound on the effective denominator degrees of freedom as

$$\begin{array}{c} df=N-K \end{array}$$

where *N* is the number of observations, and *K* is the number of all modelled fixed and random effects. No p-values were computed for the choice data as they are autocorrelated across the “potential loss” factor by design, and therefore have reduced effective numerator degrees of freedom.

We also fit models using a random interaction of the fixed effects with the subject. Models including a random threat level × potential loss interaction did not converge for most measures, indicating overparametrisation. We therefore concluded the absence of such (systematic) random interactions and report only results from models with random intercept. Note that all significant main effects on approach latency were replicated in reduced random interaction models excluding the threat level × potential loss interaction. An exception was the potential loss effect in experiments 3/4, but here the fixed-effects interaction was significant in the full fixed-effects model such that the main effects are difficult to interpret in a model removing this term.

For analysing SCR, we added to the model an additional fixed factor“Go/NoGo”and all interactions with the other two fixed factors. For analysing an impact of anxiety on approach latencies, we added an additional fixed (continuous) term“anxiety”and all interactions with the two fixed factors.

Approach and return latencies were reconstructed from the linear mixed effects model using the function lsmeans. In a nutshell, this function averages the data for each subject and experimental condition separately, and then averages over subjects, while correcting for missing values in individual subjects.

#### Comparison with the model

To reconstruct a prior on the temporal evolution of threat from the measured data, we used approach latencies reconstructed at the group level. First, we built an ideal observer model to quantify potential loss on each token occurrence. This model takes into account opportunity costs. In other words, by getting caught, one does not only lose previously collected tokens but also all additional tokens one might have collected in the remainder of the epoch. This reasoning can explain why subjects show approach delay on the first token where loss from previously collected tokens is zero. We assumed that subjects decided on a number of tokens *D*_*i*_ they desired to collect on each threat level *i*. This was estimated per subject as the average number of tokens collected on epochs on which subjects were not caught, and averaged across subjects. The potential loss of getting caught on each token was the expectation of obtaining the desired number of tokens, given that one was not caught until now and on the current trial. This took into account the number of tokens *T* collected so far, the probability *P*_*C*_ of getting caught on each future collection attempt, and the probability of obtaining a token when making a movement to collect it and not getting caught, *P*_*T*_.

$$\begin{array}{c} L_{i,T}=\left\{\begin{array}{cc} -D_{i}{\left(1-P_{C}\right)}^{\left(D_{i}-T\right)/P_{T}} & T<D_{i}\\ -T & T⩾D_{i} \end{array}\right.. \end{array}$$

We then calculated the time derivative of the expected gain for the time points of movement, to solve eq (5) for the time derivative of the prior $\bar{P}\;(t_{1})$:

$$\begin{array}{c} \frac{\partial }{\partial t_{1}}\bar{P}\left(L\right)=-\frac{\bar{P}\left(L\right)}{L_{i,T}P_{i}\left(L\right)}\frac{\partial }{\partial t_{1}}E\left(G\right) \end{array}$$

where $\bar{P}/P_{i}$ is assumed to be a constant, given by the average of the catch rates for the three threat levels divided by the individual catch rate per threat level.

These data points were then fit with a first-order polynomial using the Matlab function polyfit, integrated, and a constant determined by setting the prior to the average catch rate at the average approach delay. This fitted prior was then used to solve eq (5) for each condition, and derive the predicted approach latencies.

## Supporting Information

S1 Text Supplementary results. An example for scenario 2, a model extension incorporating metabolic costs, and additional tables and figures illustrating experimental findings.
(PDF)
