# Supplementary material for: Anxiety-Like Behavioural Inhibition Is Normative under Environmental Threat-Reward Correlations
Source: PLoS Comput Biol. 2015 Dec 9;11(12):e1004646. doi: 10.1371/journal.pcbi.1004646 (PMC4674090; doi:10.1371/journal.pcbi.1004646)
Supplement: S1 Text — An example for scenario 2, a model extension incorporating metabolic costs, and additional tables and figures illustrating experimental findings. (PDF) [file pcbi.1004646.s001.pdf]

1. Model

Table S1: Additional variables used in supplemental information (see table in main text for remaining variables)

$\widetilde{\lambda}$	Time-varying event rate of an inhomogeneous Poisson process controlling the punisher activation
λ_3	Scaling parameter of prior threat distribution
$g(t;k;\vartheta)$, $G(u;l;\vartheta)$	Pdf and cdf of a gamma distribution, describing the time-varying part of the inhomogenous Poisson process
$C(t_1)$	Biological cost of making a movement, in scenario 1 with metabolic cost

1.1. Example for scenario 2

We can model the threat events as inhomogenous Poisson process with decaying event rate, after a pellet has appeared:

$$P_{x>0} = 1 - P(x=0) = 1 - e^{-\mu(t_1,t_2)}$$

with

$$\mu(t_1,t_2) = \int_{t_1}^{t_2} \widetilde{\lambda}(u)\,du.$$

A natural predator that is activated quickly by a reward and then decreases its attention may be modelled by the sum of a constant baseline event rate and the pdf of a Gamma distribution $g(t;k;\vartheta)$ with cdf $G(u;l;\vartheta)$. For illustration, we choose a mode of the distribution at 0.4 s and a fixed relation of baseline rate and time-varying rate:

$$\widetilde{\lambda}(t) = \lambda_3\left(0.1 + 0.9g(t;5;0.1)\right).$$

$$\mu(t_1,t_2) = \left[0.1\lambda_3 u\right]_{t_1}^{t_2} + \left[0.9\lambda_3 G(u;5;0.1)\right]_{t_1}^{t_2}$$

$$= 0.1\lambda_3\Delta t + 0.9\lambda_3\left(G(t_2;5;0.1) - G(t_1;5;0.1)\right).$$

Both $E(G)$ and $E(L)$ explicitly depend on t_1, and for the given prior, the expected outcome is maximised for some $t_1^* > 0$. For the illustrations in Fig. 1/2 of the main text, the following assumptions were made:

The animal correctly learns the threat probability of scenario 1 over the interval $[t_1,t_2]$, but believes it is in scenario 2 and therefore assigns them to the wrong underlying process, i. e. $\mu(t_1,t_2) = \lambda_2$. Further, we assume that the animal in all trials moves at $t_1 = 1\,s$ with $\Delta t = 1\,s$. Then we have

$$\mu(t_1,t_2) = 0.1\lambda_3\Delta t + 0.9\lambda_3\left(G(t_2;5;0.1) - G(t_1;5;0.1)\right) = \lambda_2\Delta t.$$

From the above, it follows that

$$\lambda_3 = \lambda_2 \Delta t / \left(0.1\Delta t + 0.9G\left(t_2; 5; 0.1\right) - 0.9G\left(t_1; 5; 0.1\right)\right) \cong 7.9169\lambda_2.$$

For this example, the average shock rate is $p = 0.1$ for each move. This corresponds to $\lambda_2 = 0.1054/s$, such that $\lambda_3 = 0.8341$. Together with an expected pellet withdrawal time of 1.25 s, corresponding to $\lambda_1 = 0.8$, and $G = 1$, $L = 2$, these parameters were used for the illustrations in figure 1/2, and result in an optimal approach latency of 1.01 s.

1.2. Impact of metabolic cost in scenario 1

This is equivalent to scenario 1 with one additional assumption: There is a biological cost of moving which explicitly depends only on t_1.

This could arise, for example, because moving early when a food pellet appears requires the animal to be in state of higher arousal and has metabolic cost. In this scenario:

$$Z = E\left(G\right) + E\left(L\right) + C\left(t_1\right).$$

Analoguous to scenario 2, there exists a non-zero maximiser t_1^* for Z if $C\left(t_1\right)$ fulfills the conditions specified for $E\left(L\right)$ in scenario 2.

1.3. Impact of parameter changes in scenario 1 with metabolic cost

If a non-zero maximiser t_1^* exists, we must have

$$\frac{\partial}{\partial t_1} Z = \frac{\partial}{\partial t_1} E\left(G\right) + \frac{\partial}{\partial t_1} C\left(t_1^*\right) = 0.$$

Analogous to the argument made for scenario 2, we have

$$t_1' - t^*{}_1 = -\frac{\frac{\partial}{\partial t_1} C\left(t_1^*\right) + G\frac{\partial}{\partial t_1} P_G\left(t_1^*\right)}{\frac{\partial^2}{\partial t_1^2} C\left(t_1^*\right) + G\frac{\partial^2}{\partial t_1^2} P_G\left(t_1^*\right)}.$$

This demonstrates that $t_1' - t_1^*$ is sensitive to changes in G but not to changes in L or $E\left(L\right)$.

2. Supplemental results

Table S2 (next page): Results from a 3 ×5 or 2 ×4 Linear Mixed Effects Model on choice, approach latency, return latency, and correctness of response. No exact p-values can be computed for choice data (see methods). In experiment 4, the time point of leaving the safe place was chosen to compute approach latency. See table S2 for the initiation of movement which shows the same pattern.

	Choice		Approach latency			Return latency			Correctness of response		
	F	*df*	*F*	*df*	*p*	*F*	*df*	*p*	*F*	*df*	*p*
**Experiment 1**											
Threat level	12.6	2, 15923	22.4	2, 15493	< 5e-10	31.9	2, 12788	< 5e-14	< 1	2, 15734	n. s.
Threat level: linear			21.9	1, 15493	< 5e-6	35.1	1, 12788	< 5e-9			
Potential loss	785.9	5, 15923	12.4	4, 15493	< 5e-10	14.2	4, 12788	< 5e-11	< 1	4, 15734	n. s.
Potential loss: linear			19.8	1, 15493	< 1e-5	22.3	1, 12788	< 5e-6			
Threat level × potential loss	2.14	10, 15923	< 1	8, 15493	n. s.	< 1	8, 12788	n. s.	2.1	8, 15734	< .05
Interaction: linear × linear			< 1	1, 15493	n. s.	< 1	1, 12788	n. s.			
**Control experiment 2**											
Threat level	4.1	2, 13228	81.7	2, 11808	< 1e-35	25.3	2, 9910	< 1e-10	3.6	2, 11905	< .05
Threat level: linear			38.9	1, 11808	< 5e-10	2.2	1, 9910	n. s.			
Potential loss	571.9	5, 13228	19.5	4, 11808	< 1e-15	5.1	4, 9910	< .001	2.4	4, 11905	= .05
Potential loss: linear			7.9	1, 11808	< .01	7.7	1, 9910	< .01			
Threat level × potential loss	< 1	10, 13228	4.3	8, 11808	< 1e-4	1.1	8, 9910	n. s.	1.5	8, 11905	n. s.
Interaction: linear × linear			6.1	1, 11808	< .05	1.6	1, 9910	n. s.			
**Experiment 3**											
Threat level	7.6	2, 17001	26.4	2, 17398	< 5e-12	31.2	2, 14317	< 5e-14	< 1	2, 17505	n. s.
Threat level: linear			21.9	1, 17398	< 5e-6	20.4	1, 14317	< 1e-5			
Potential loss	781.3	5, 17001	12.1	4, 17398	< 1e-9	9.3	4, 14317	< 5e-7	3.2	4, 17505	< .05
Potential loss: linear			13.0	1, 17398	< 5e-4	9.4	1, 14317	< .005			
Threat level × potential loss	< 1	10, 17001	2.7	8, 17398	< .01	< 1	8, 14317	n. s.	< 1	8, 17505	n. s.
Interaction: linear × linear			< 1	1, 17398	n. s.	< 1	1, 14317	n. s.			
**Experiment 1 and 3 combined**											
Threat level	19.8	2, 32923	41.9	2, 32902	< 1e-18	60.8	2, 27116	< 1e-26	< 1	2, 33250	n. s.
Potential loss	1558.5	5, 32923	12.2	4, 32902	< 1e-9	29.4	4, 27116	< 1e-23	1.7	4, 33250	n. s.
Experiment	< 1	1, 32923	587.9	1, 32902	< 1e-128	59.4	1, 27116	< 1e-13	1.1	1, 33250	n. s.
Threat level × potential loss	2.8	10, 32923	1.2	8, 32902	n. s.	< 1	8, 27116	n. s.	1.0	8, 33250	n. s.
Threat level × experiment	2.6	2, 32923	2.8	2, 32902	= .06	2.2	2, 27116	n. s.	< 1	2, 33250	n. s.
potential loss × experiment	7.8	5, 32923	7.1	4, 32902	< 1e-4	1.2	4, 27116	n. s.	2.0	4, 33250	= .09
Threat level × token no. × exp.	< 1	10, 32923	2.1	8, 32902	< .05	< 1	8, 27116	n. s.	1.4	8, 33250	n. s.
**Experiment 4**											
Threat level	3.4	2, 14885	16.5	2, 14631	< 1e-7	29.5	2, 11734	< 1e-12	< 1	2, 14715	n.s.
Threat level: linear			13.2	1, 14631	< 5e-4	34.5	1, 11734	< 5e-9			
Potential loss	685.1	5, 14885	3.0	4, 14631	< .05	78.3	4, 11734	< 1e-64	< 1	4, 14715	n.s.
Potential loss: linear			3.7	1, 14631	= .05	63.2	1, 11734	< 5e-15			
Threat level × potential loss	1.3	10, 14885	2.1	8, 14631	< .05	< 1	8, 11734	n. s.	< 1	8, 14715	n.s.
Interaction: linear × linear			< 1	1, 14631	n. s.	< 1	1, 11734	n. s.			

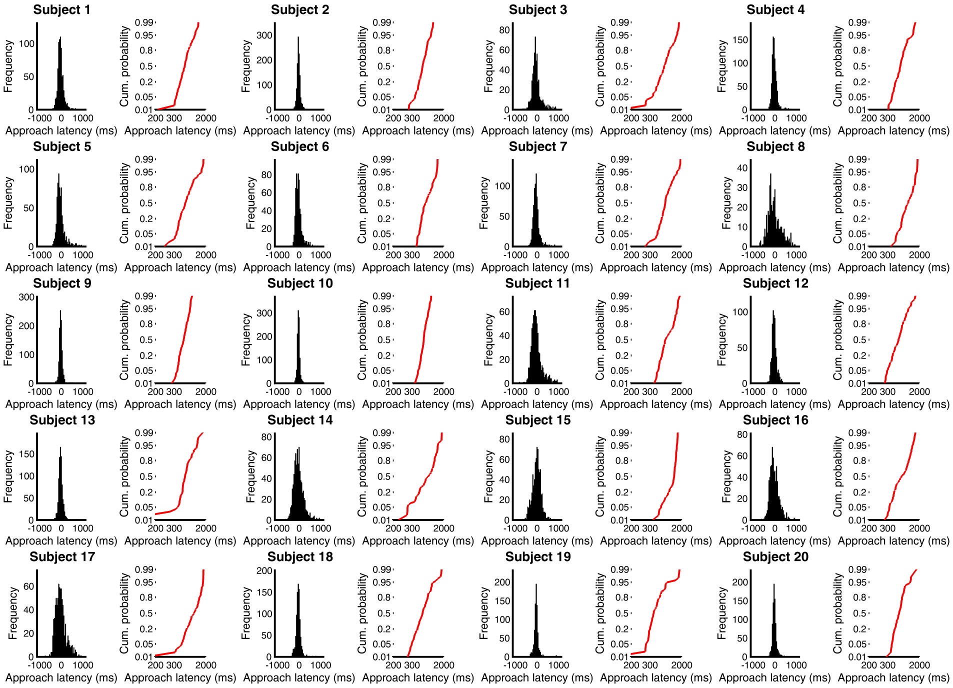

Fig. S1: Approach latency distributions for experiment 1. For each participant, the left plot shows the distribution of deviations from the condition mean which is, for most participants, rather peaked. Decision processes in the absence of sensory evidence accumulation are often assumed to result in a drift to threshold with rate that is constant within one trial and normally distributed across trial, reflected in a recinormal reaction time distributions (LATER model, Carpenter & Williams 1995). The right plot for each participants shows a reciprobit plot for approach latencies from the first condition (1st token, low threat). This is clearly non-linear for almost all participants, in particular also for those with heavy-tailed approach latency distributions. Thus there is no evidence that the distribution of approach latencies is due to an underlying evidence accumulation process.

Table S3 (next page): Results from a 2 ×4 Linear Mixed Effects Models on maximum joystick excursion in NoGo trials (i. e. subthreshold responses), motor initiation latency and response duration until leaving the safe place in Go trials, and estimated phasic sympathetic arousal, for experiment 4. Estimated sympathetic arousal was larger for NoGo trials and for higher potential loss, and the latter effect was more pronounced on NoGo trials. See Fig. S2 for reconstruction of the other measures.

	Maximum joystick excursion (NoGo)			Motor initiation latency (Go)			Response duration (Go)		
	F	*df*	*p*	*F*	*df*	*p*	*F*	*df*	*p*
**Experiment 4**									
Threat level	8.7	2, 7393	< .001	28.3	2, 14553	< 1e-12	< 1	2, 14553	n. s.
Threat level: linear	15.7	1, 7393	< 1e-4	16.3	1, 14553	< 1e-4	< 1	1, 14553	n. s.
Potential loss	27.2	4, 7393	< 1e-21	12.6	4, 14553	< 1e-9	5.3	4, 14553	< .001
Potential loss: linear	62.1	1, 7393	< 1e-14	8.7	1, 14553	< 5e-3	2.1	1, 14553	n. s.
Threat level × potential loss	< 1	8, 7393	n. s.	2.8	8, 14553	< .01	1.2	8, 14553	n. s.
Interaction: linear × linear	1.8	1, 7393	n. s.	< 1	1, 14553	n. s.	< 1	1, 14553	n. s.

	Phasic sympathetic arousal		
	F	*df*	*p*
**Experiment 4**			
Go/NoGo	102.3	1, 568	<1e-21
Threat level	< 1	2, 568	n. s.
Potential loss	5.6	5, 568	<1e-4
Threat level × potential loss	< 1	10, 568	n. s.
Go × threat	< 1	2, 568	n. s.
Go × token	2.4	5, 568	< .01
Go x threat x token	2.4	10, 568	< .01

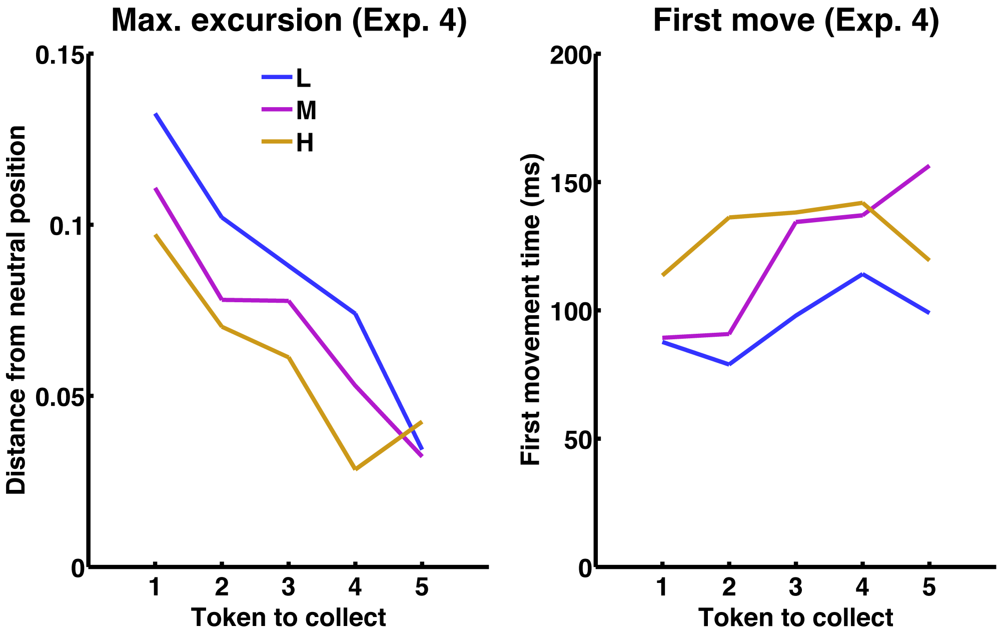

Fig. S2: Experiment 4. Left: Maximum joystick excursion on NoGo trials. Right: Latency (im ms) of motor initiation. The plots show reconstructed cell means, accounting for unequal distribution of data points.

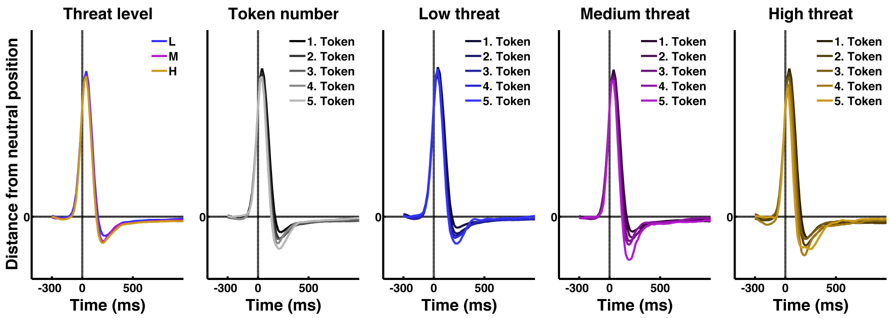

Fig. S3: Experiment 4. Absolute joystick movement (signed vector modulus towards or away from token), centred on the time point when the player left the safe place.
